# Trauma unit management and outcomes at an urban tertiary hospital in sub-Saharan Africa: a descriptive study

**DOI:** 10.4314/ahs.v21i4.36

**Published:** 2021-12

**Authors:** Tonny Stone Luggya, Annet Alenyo Ngabirano, Sarah Richardson, John Osire, Lilian Achieng, Josephine Nabulime, Jackie Mabweijano

**Affiliations:** 1 Department of Anaesthesia and Critical Care, College of Health Sciences, Makerere University; 2 Department of Emergency Medical Services, Ministry of Health Uganda; 3 Emergency Medicine Department, University of Edinburgh; 4 Accident and Emergency Department, Directorate of Surgical services, Mulago Hospital

**Keywords:** Trauma, trauma care, emergency care, head injury

## Abstract

**Background:**

Injuries are a neglected burden despite accounting for 9% of deaths worldwide which is 1.7 times that of hiv, tb and malaria combined. Trauma remains overlooked as research and resources are focused on infectious diseases. Uganda with limited trauma epidemiological data has one of the highest traumatic injury rates. This study describes demographics, management and outcomes of patients admitted to mulago hospital trauma unit.

**Materials and methods:**

This study was a retrospective record review from july 2012 to december 2015. A data collected included age, time and vitals of admission plus interventions, management and outcomes after which it was analyzed.

**Results:**

834 patient records were reviewed. The predominant age group was 18–35 and 86% of the patients were male. 54% of the patients presented during day and majority of the admission had gcs of less than 8. Antibiotics were given to 467 patients with mechanical ventilation (301) and intubation (289) as the frequent interventions done. 52% of admitted patients were discharged and 40% died.

**Conclusion:**

Most admissions' were of youthful age and had severe head injuries (gcs<8). 56% received antibiotics with frequent interventions beig mechanical ventilation and intubation. 52% of admitted patients were discharged and 40% died

## Background

Injuries are a neglected epidemic worldwide, with the seminal Global Burden of Disease (GBD) framework in 1990 estimating that injuries accounted for more than 15% of all ill-health in the world with a forecast to increase to 20% by 2020 1. By 2030 road accidents will be a leading cause of death, in Low and Middle Income Countries (LMICs), ahead of Malaria, Tuberculosis and H.I.V, yet already globally 90% of the related deaths and disabilities due to trauma occur predominantly in LMICs 2. Also With a projected 40% increase in global deaths due to injury, the WHO predicts that just one type of injuries, Road Traffic Accidents (RTAs), will rise from the 9th to the 3rd highest cause of world burden of disease by 2030 2.

Uganda is a low-income country in sub-Saharan Africa With reported number of fatal crashes in Uganda has increased 7-fold in 25 years, from 500 in 1991 to 3,503 in 2016 according to annual Police Report Data 3. Trauma in Uganda is a killer of epidemic proportions injuries due to lack of a proper surveillance system are less appreciated and therefore have been given low priority and little or no resources 4. Also concerning rate of fatality, a further 12,754 serious injuries from crashes were reported in 2013^3^. The estimate of the actual fatality may significantly higher if all injuries were formally recorded, however the crude rate in Uganda, is the Police annual crime and traffic/road safety report, showed that in 2013 we had 18,368 crashes out of which 14.2% were fatal and 48.3% were serious 5. The economic impact of road crashes on the overall annual cost to the health system and the economy of was estimated at approximately $1.2 billion, equivalent to 5% of Uganda's gross domestic product (GDP) 3. The economic and health impact of injury of other causes such as assault and falls remains unknown.

Kampala, the capital city of Uganda, is faced with road traffic injuries as the largest cause of both morbidity and mortality by far across all ages mainly affecting the school-aged children 6.

Mulago hospital, located in Kampala, is Uganda's National Referral and Makerere University teaching hospital, that's faced with staff issues like lack of motivation and professionalism due to poor pay coupled with overcrowding, a poorly functioning referral system, limited quality assurance and a cumbersome procurement system^7^.

Despite being the main center for Trauma referrals for the country, the true extent of the Trauma injury admission burden and outcome at the facility is largely unknown as there is a lack of accurate data available related to the actual utilization of the health services provided.

If health policy makers are to address the critical burden of injury in Uganda, they must first understand the outputs and outcomes of the patients presenting following trauma. The rationale of this study was thus to retrospectively determine the management and outcomes of the patients admitted to the Shock Trauma Unit from its inception to over a 3 year period. This study therefore set out to assess the units' impact in the Accident and Emergency (A&E) Department of Mulago National Referral and Teaching Hospital (MNRTH).

## Materials and methods

### Study design

we conducted a 3-year retrospective record review and descriptive study

### Ethical Approval

Ethical approval was obtained from the Department of Anaesthesia and Critical Care, Makerere University School of Medicine Internal Review Board (SOMREC) and MNRTH IRB

### Study Setting

MNRTH is a 1500 bed tertiary hospital caring for approximately 140,000 patients annually, 48,000 of which transition through the A&E with trauma related injuries^8^. The Shock Trauma Unit was comprised of 6 beds, 2 capable of delivering short-term mechanical ventilation (level 3 care) and 4 capable of High Dependency care delivery (level 2 care). The Unit was supported technically by the Department of Anesthesia, it was staffed with 2 Specialist Doctors (1 Surgeon and 1 Anaesthesiologist) and 10 nurses, with an average nurse to patient ratio of 1:4. The Shock Trauma Unit received critical level care patients requiring immediate stabilization for ICU care, theatre procedures or where additional specialist input is required prior to ward admission.

### Study periods

Retrospective patient records and charts were reviewed from the period of the Trauma Unit creation in July 2012 to the end of December 2015.

### Participants

All admissions in the shock Trauma Unit for the study period with retrievable data or records

### Study variables

The main variables of interest were admission demographics, baseline vitals, treatment options and management given, patient outcomes and disposition. And the data source was the patients' admission records and chart reviews.

### Data management and analysis

A structured pretested and validated questionnaire was used as the data collection tool. All patient records were considered eligible for inclusion in the data set, with no exclusion criteria being applied other than complete lack of a patient record that was unable to offer data enough to be used for analysis. Confidentiality and addressing bias was done by allocating unique study numbers for each patient to allow de-identifying of data at the collection point. Once collected, data was cleaned and missing data confirmed or rectified then compiled into a database using Epi DATA and statistical analysis performed using Microsoft Excel and Prism version 8.0.

## Results

We collected data of eight hundred and thirty four (834) available patient records that were potentially eligible, examined for eligibility and eventually analyzed. The completely missing data or records were not considered.

### Demographics

**Figure 1:**
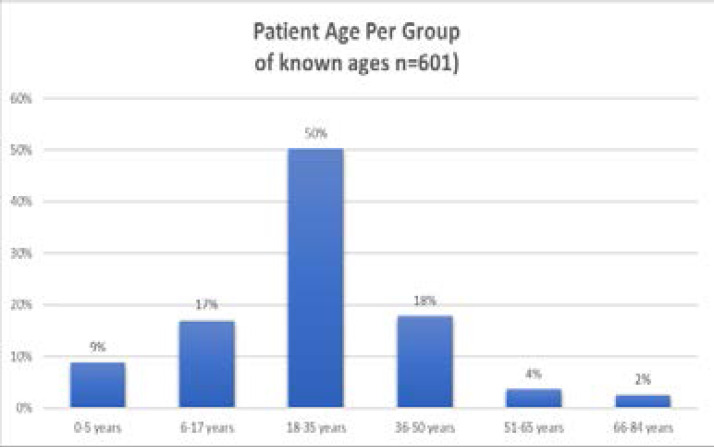
Admitted patients age distribution

Of the 834 patient admission records, 86% were male and 14% were female, with 5 (0.5%) having no gender documented. Age was documented in 601 out of 834 patients (72%). Of those with a documented age (n=601), 50% were between 18–35, with 17% aged 6–17 and 9% under 5. Those with undocumented age were 28% (n=233). Age ranged from 0–84 years. See figure 1.

### Admission Times

Admission time was documented in 592 cases, it was unknown in 242 cases. Documented admission times showed most patients 54% (n=317) presented at daytime, 24% (n=133) cases came at night and 22% (133) came in the evening.

### Admission GCS

**Figure 2:**
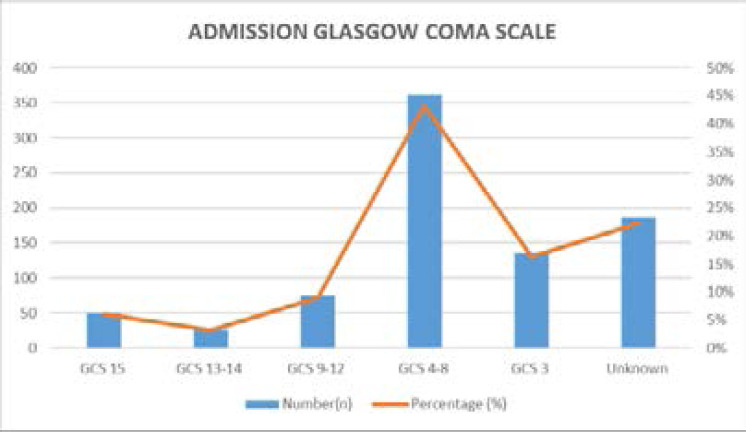
Patients admission glasgow comma SCALE (GCS)

Admission GCS was documented in 648 cases, with majority (43.3%) being between GCS 4–8. 186 cases (22.3%) had no documented admission GCS. See figure 2

### Admission spo2

Majority of the admissions SPO2 were between 95–100%. 195 (23.4%) didn't have an admission SPO2 taken. See table 1

**Table 1:** Admission SPO2

SPO2	Number (n)	Percentage (%)
95–100	385	46.2 %
90–94	102	12.2 %
80–89	65	7.8 %
70–79	31	3.7 %
60–69	19	2.3 %
50–59	16	1.9 %
30–49%	21	2.5 %
Unrecorded	195	23.4 %

### Other measured parameters

Temperature was recorded in 7 patients only

Respiratory Rate (RR) was unrecorded in 820 patients & the recorded ones were

- RR 10–20 in 6
- RR of 21–29 in 2
- RR of 30–39 in 1
- RR 40–47 in 5

### Interventions and tests done in the trauma unit

**Figure 3:**
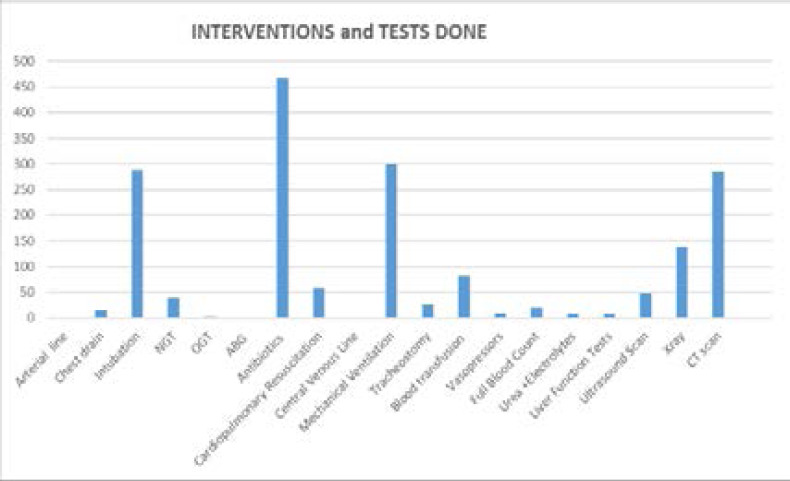
Interventions and tests done in the trauma unit *(total > 834 due to cross interventions in multiply injured patients*)

Of the documented interventions done; 467 got antibiotics, 301 were mechanically ventilated and 289 were intubated as the third frequent intervention done followed by Computed Tomography (CT) scan in 286 cases as the fourth commonest intervention as shown in figure 3.

### Critical events in the trauma unit

**Figure 4:**
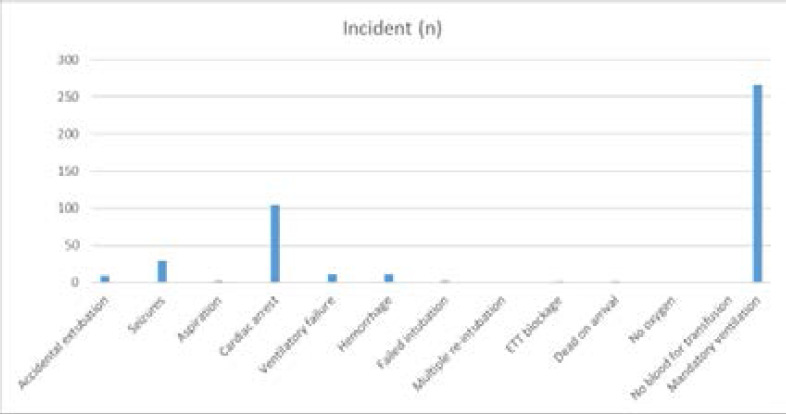
Critical incidents documented in the trauma unit

Mandatory ventilation (267) was the most critical event reported, this was followed by cardiac arrests (105) then seizures (29). See figure 4

### Disposition of admitted patients

**Figure 5:**
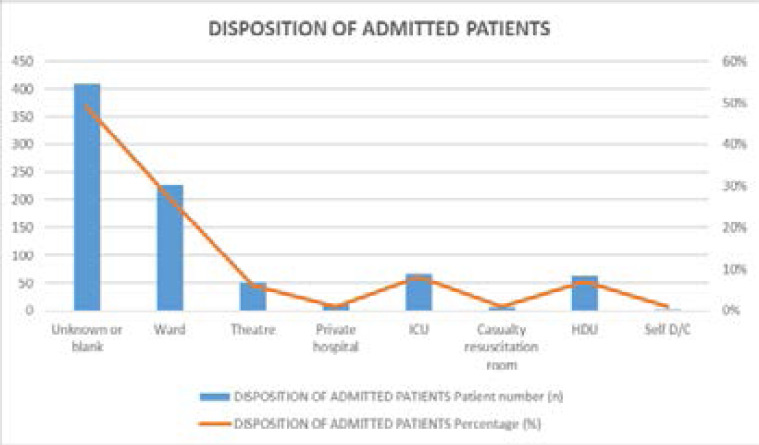
Disposition of patients after trauma unit management

408 (49%) had undocumented disposition, while for the documented cases majority(27%) were transferred to the ward, followed to ICU (8%) then thirdly to theater (6%) as elaborated in figure 5

### Outcomes of admitted patients

52% of admitted patients were discharged and 40% died, with 8% having unknown (undocumented) outcome.

## Discussion

This three-year retrospective study is the first of its kind on the patients admitted to the MNRTH shock trauma unit that received the highest acuity level of patients in the A&E. This data provides a true understanding of the trauma burden received at the national referral facility in Uganda. Notably studies have shown that by 2030 injury will be one of the top 5 the leading causes of death globally 9. Our study showed that majority of injury admissions are of the youthful age group, meaning we shall continuously experience trauma related injury and death as demographic national data shows that the majority of Uganda's population is youthful with 95% between 0–54 years of age and 48% aged 0–14 10. This is further compounded by Uganda's explosive growth in the numbers of bicycles and motor cycles pluboth fast motorized traffic and vulnerable road users on the roads 3, 4.

This study also showed that majority of the patients admitted had Severe Head Injury with majority having admission GCS of 4–8 which complicates management plus prognosis if the golden hour window is lost and this may explain our studies relatively high mortality of 40%. The severity of trauma presenting poses a challenge because already local studies have shown that Kampala is faced with RTAs as the largest cause of both morbidity and mortality by far across all ages mainly affecting the school-aged children 6, with 65% of injured patients dying from Head and Neck injuries in Kampala^11^. This is further compounds timely and holistic Trauma care offered as local reports have showed that MNRTH “chokes-on-accident-victims &costs” 12. This was thought to be remedied by the Israel government donation of the shock Trauma Unit to Mulago Hospital in 2012 to reduce the high Trauma case load of about 60–100 patients a day 13.

Poor or complete patient documentation was highlighted as a glaring issue by this study as evidenced by the primary admission vitals commonly recorded being GCS and SPO2. We posit that despite the Israel government donation, MNRTH was already faced with shortages and system challenges affecting health worker motivation. We also postulate that complete documentation may have been compounded by overwhelming numbers and glaring staff shortages with studies showing a nurse: patient ratio of 1:40 at a given time^14^. This we think was reflected in the high unknown disposition of almost half of the patients after initial stabilization in the unit, though also some of the patients lacked all information because commonly where details are not recorded especially for “john Doe's” the patient is registered as ‘unknown’. We think this will be a long standing challenge that MNRTH management has to tackle too because local studies have shown that lack of supplies, overwhelming number of patients, and inadequate staffing also interfere with consistent monitoring and documentation of patient's records 15. This we think may have affected crucial timely interventions that needed to be done hence contributing to the critical events that occurred like seizures and cardiac arrests which eventually had an impact on outcomes of study patient's managed in the unit.

We think this trend can be reversed as there is increasing evidence that implementing a robust trauma registry and injury surveillance system facilitates evidence-based resource distribution 16. Also because studies have shown promise that a hospital based registry, if well instituted, can open the opportunity to replicate the process elsewhere in a similar context and hopefully feed into the global agenda of reducing deaths and disabilities from Traumatic Brain Injuries (TBI) in low-and middle-income countries 17.

This study will hopefully through data capture and analysis enlighten on the need for true utilization and understanding of an emergency trauma service. Also it can enable policy makers address critical issues and adequate resource allocation to MNRTH to aid in reducing the morbidity and mortality experienced by Ugandans.

## Conclusion

Majority of the trauma unit admissions' were of youthful age and had severe head injuries with GCS less than 8. Antibiotics were given to more than half of the admissions, followed by intubation and Mechanical Ventilation as commonest interventions done. Majority of the admissions had “undocumented’ disposition and 52% discharge outcomes plus Mortality of 40%

## List of abbreviations

MNRTH: Mulago National Referral and Teaching Hospital

A&E: Accident and Emergency

RTAs: Road Traffic Accidents

GCS: Glasgow Comma Scale

TBI: Traumatic Brain Injury

## References

[1] Lopez A, Mathers C, Ezzati M, Jamieson D, Murray C (2006). Global burden of disease and risk factors.

[2] Mathers C D, Loncar D (2006). Projections of global mortality and burden of disease from 2002 to 2030. PLoS Med.

[3] United Nations Economic Commission for Africa (2018). Road Safety Performance Review Uganda.

[4] Andrews CN, Kobusingye OC, Lett RR (1998). Road traffic accident injuries in Kampala. East Afr Med J.

[5] (2013). Uganda Police Annual Crime and Traffic/ Road Safety Report.

[6] Hsia R Y, Ozgediz D, Mutto M, Jayaraman S, Kyamanywa P, Kobusingye O C (2010). Epidemiology of injuries presenting to the national hospital in Kampala, Uganda: Implications for research and policy. Int J Emerg Med.

[7] Kizza IB, Tugumisirize (2011). Makerere University College of Health Sciences' role in addressing challenges in health service provision at Mulago National Referral Hospital. BMC Int Health Hum Rights.

[8] Tran TM, Fuller AT, Kiryabwire J, Mukasa J, Muhumuza M, Ssenyojo H, Haglund MM (2015). Distribution and characteristics of severe traumatic brain injury at Mulago National Referral Hospital in Uganda. World Neurosurg.

[9] Vavilala M, Curry P, Ramaiah R (2011). Current trends and update on injury prevention. Int J Crit Illn Inj Sci.

[10] https://unstats.un.org/unsd/demographic/sources/census/wphc/Uganda/UGA-2016-05-23.pdf.

[11] Jayaraman S (2011). Disparities in injury mortality between Uganda and the United States: comparative analysis of a neglected disease. World Journal of Surgery.

[12] http://kfm.co.ug/health/mulago-hospital-chokes-on-accident-victims-costs.html.

[13] Ayebazimbwe Agatha, Ssenoga Brian Mulago hospital to get trauma center, Daily Monitor.

[14] Ozgediz D G M (2008). neglect of the global surgical workforce:Experience and evidence from Uganda. World Journal of Surgery.

[15] Wynveen L, Gamble M, Nabulime J, Luggya T, Kalanzi J K, Mowafi H (2018). A qualitative study exploring nurses' attitudes, confidence, and perceived barriers to implementing a traumatic brain injury nursing chart in Uganda. African J Emerg Med.

[16] Kisitu D K (2016). A pilot orthopedic trauma registry in Ugandan district hospitals. J Surg Res.

[17] Mehmood A, Zia N, Hoe C, Kobusingye O, Ssenyojo H, Hyder AA (2018). Traumatic brain injury in Uganda: exploring the use of a hospital based registry for measuring burden and outcomes. BMC Res Notes.

